# Hypothyroidism correlates with osteoporosis: potential involvement of lipid mediators

**DOI:** 10.3389/fmed.2024.1453502

**Published:** 2024-11-01

**Authors:** Pengyuan Leng, Ying Qiu, Mengxue Zhou, Yuhang Zhu, Na Yin, Mingming Zhou, Weili Wu, Min Liu

**Affiliations:** ^1^Department of Orthopedics, The Third Affiliated Hospital of Wenzhou Medical University, Wenzhou, Zhejiang, China; ^2^Postgraduate Training Base Alliance of Wenzhou Medical University (The Third Affiliated Hospital of Wenzhou Medical University), Ruian, Zhejiang, China; ^3^College of Nursing, Hangzhou Normal University, Hangzhou, China; ^4^Department of Anesthesiology, The Third Affiliated Hospital of Wenzhou Medical University, Wenzhou, Zhejiang, China; ^5^Department of Thyroid and Breast Surgery, The Third Affiliated Hospital of Wenzhou Medical University, Wenzhou, Zhejiang, China

**Keywords:** circulating metabolites, Mendelian randomization, mediation analysis thyroid dysfunction, osteoporosis, thyroid-related hormones

## Abstract

**Background:**

Observational studies have demonstrated a correlation between thyroid dysfunction and osteoporosis (OP); however, the underlying causality has yet to be fully elucidated.

**Methods:**

The necessary dataset was sourced from public databases. Initially, instrumental variables (IVs) were selected based on three primary hypotheses. Subsequently, Cochran’s Q test was employed to exclude IVs exhibiting heterogeneity. The MR-PRESSO test and the leave-one-out sensitivity test were further applied to detect potential pleiotropy. Inverse variance was utilized for the analysis. This study primarily utilized the inverse variance weighted (IVW) model for Mendelian analysis. Since Type 1 diabetes mellitus can also contribute to the development of osteoporosis, this study additionally employed multivariate Mendelian analysis. Furthermore, 249 circulating metabolites were selected for mediation analysis in the Mendelian randomization framework.

**Results:**

In this study, the two-sample Mendelian randomization (MR) analysis primarily employed the random-effects IVW model and demonstrated a causal relationship between hypothyroidism (OR = 1.092, 95% CI: 1.049–1.137, *p* < 0.001) and hyperthyroidism (OR = 1.080, 95% CI: 1.026–1.137, *p* = 0.003) with the risk of OP. No causal relationships were identified between FT3, FT4, TSH, and the risk of OP (*p* > 0.05). The results of the multivariate Mendelian randomization (MVMR) analysis indicated that hyperthyroidism was no longer a risk factor for OP (OR = 0.984, 95% CI: 0.918–1.055, *p* = 0.657), whereas hypothyroidism persisted as a risk factor (OR = 1.082, 95% CI: 1.021–1.147, *p* = 0.008). The mediated Mendelian randomization analysis revealed that hypothyroidism may exert an indirect effect on OP via triglycerides in large VLDL, mediating approximately 2.47% of the effect.

**Conclusion:**

This study identifies a potential link between hypothyroidism and OP, possibly mediated indirectly via triglyceride levels in large VLDL. Further investigations are required to elucidate the direct or indirect causal mechanisms underlying this association.

## 1 Introduction

American scholars project that by 2025, more than 3 million fractures will occur among osteoporosis patients in the United States, with associated medical costs expected to reach $25.3 billion (1). Osteoporosis (OP), a hallmark of aging, is a systemic metabolic bone disease characterized by decreased bone mineral density and increased fracture susceptibility, and, from a cytological perspective, by an imbalance in the functions of osteoblasts, osteoclasts, and adipocytes (2, 3). Multiple factors contribute to the development of OP, including insufficient calcium intake, smoking, alcohol consumption, long-term use of certain medications, and the presence of specific diseases (4).

Hyperthyroidism and hypothyroidism are the most prevalent thyroid dysfunctions, each influencing various physiological functions. In the United States, hypothyroidism affects approximately 5% of the population, while hyperthyroidism affects nearly 1% (5). Numerous observational studies have demonstrated an association between thyroid dysfunction and OP (6); however, the causal relationships between hyperthyroidism, hypothyroidism, TSH, FT4, FT3, and OP remain unclear.

To elucidate the causal relationship, Mendelian randomization (MR) analysis was conducted in this study. MR analysis is a widely used method that evaluates potential causal relationships between exposure factors and outcomes using instrumental variables (IVs) and is less susceptible to confounders, thereby producing more reliable results (7). Therefore, this study employed MR analysis to investigate the potential causal relationship between thyroid dysfunction, thyroid function, and the risk of OP.

Patients with type 1 diabetes have a higher likelihood of developing thyroid dysfunction, and type 1 diabetes is also a known risk factor for OP. Therefore, this study incorporated type 1 diabetes into the multivariate Mendelian randomization (MVMR) analysis (8). This was done to explore the effect of adjusting for type 1 diabetes on OP, and subsequently to assess whether a causal relationship exists between thyroid dysfunction and OP.

Metabolomics is an emerging field of research that offers novel insights into how metabolites influence various physiological systems, particularly the endocrine system (9). The metabolome consists of metabolites and their associated metabolic pathways (10). A study analyzed plasma metabolites from 41 individuals with subclinical hypothyroidism, 45 hypothyroid patients, and 40 euthyroid individuals using metabolomics and machine learning algorithms. The findings revealed that the metabolic patterns of the hypothyroid and subclinical hypothyroid groups differed significantly from those of the euthyroid group. Specifically, primary bile acid biosynthesis, lysine degradation, tryptophan metabolism, steroid hormone biosynthesis, and purine metabolism were significantly impacted (11). However, few studies have focused on circulating metabolites in plasma.

Several observational studies have demonstrated an association between hypothyroidism and dyslipidemia, as well as a link between dyslipidemia and OP (12, 13). However, it remains unclear whether hypothyroidism influences the development of OP through circulating metabolites. Therefore, this study will explore this question using mediated Mendelian randomization analysis.

## 2 Methods and materials

### 2.1 Study design and data sources

**Figure 1:**
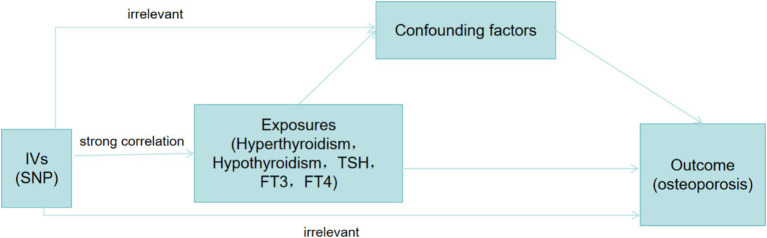
Diagram for this study. IVs, instrumental variable; SNP, single nucleotide polymorphism.

Our data were sourced from the widely recognized IEU OpenGWAS database, the ThyroidOmics Consortium, and the FinnGen database (Table 1). As these databases were externally developed, ethical approval was not required. In this study, hyperthyroidism, hypothyroidism, TSH, FT4, and FT3 were used as exposure variables, with OP as the outcome variable. An MR analysis was conducted to investigate potential causal relationships between these factors (Figure 1).

**Table 1:** Sources and status of data.

Exposures/outcome	Sample size	Data sources	Ancestry	GWAS ID	Total SNPs	Selected SNPs
Hyperthyroidism	46	IEU open database	European	ebi-a-GCST90018860	24,189,279	11
Hypothyroidism	405,357	IEU open database	European	ebi-a-GCST90013893	11,038,721	104
TSH	271,040	The ThyroidOmics Consortium database	European	–	–	161
FT3	59,061					
					–	6
FT4	119,120					
					–	60
Osteoporosis	399,054	Finn Biobank	European	R10_M13_OSTEOPOROSIS	–	–

### 2.2 Instrumental variables

In MR analysis, instrumental variables are genetic variants, often single nucleotide polymorphisms (SNPs), used to study the impact of genetic variation (14). The screening criteria were as follows: (1) instrumental variables must be strongly correlated with exposure variables. We set a significance threshold of *p* < 5 × 10^-8 to identify SNPs with strong associations. (2) Instrumental variables must be independent, meaning they are not correlated with confounders (15). Linkage disequilibrium among SNPs was addressed by setting kb = 10,000 and *r*^2^ ≤ 0.001 (16). (3) Instrumental variables should affect the outcome exclusively through the exposure variables (17). The *F*-statistic assesses the strength of the correlation between instrumental variables and exposure factors. Thus, we calculated the *F*-statistic for each exposure-related SNP (*F* = *β*^2^/SE^2^) (18), where *β* represents the effect estimate of the exposure factor and SE is the standard error. We selected IVs with *F* > 10 to reduce bias from weak instruments (19). Subsequently, we identified SNPs where the exposure and outcome overlapped to remove palindromic SNPs and misaligned alleles.

### 2.3 MR analysis

To determine whether a causal relationship exists between genetic exposure and outcome, we employed several commonly used MR methods, including inverse-variance weighted (IVW), MR-Egger regression, weighted median (WM), weighted mode techniques, and MR-PRESSO. IVW is widely regarded as the primary statistical method in MR, and thus, this study adopts IVW as the main analysis approach. The fixed-effects IVW model operates under the assumption that all instrumental variables (IVs) are valid, whereas the random-effects IVW model assumes that not all IVs are valid (20). Therefore, we initially conducted a heterogeneity test using R. If heterogeneity was detected (i.e., *p* < 0.05), the random-effects model was applied (21). Subsequently, a pleiotropy test was conducted, and if *p* > 0.05, it indicated that the likelihood of pleiotropy was minimal or nonexistent and could be disregarded (22). Additionally, the intercept of the MR-Egger regression was not statistically significant, and the funnel plot was symmetric around zero, further confirming the absence of pleiotropy. A Cochran Q test was also conducted to assess SNP heterogeneity, which was statistically significant at *p* < 0.05 (23).In this study, we used the “TwoSampleMR,” “MendelianRandomization,” and “MR-PRESSO” packages in R (version 4.4.0) for MR analysis (24).

### 2.4 Mediator analysis link “hypothyroidism-blood-metabolites-OP”

Data on 249 circulating metabolites, collected and provided by Nightingale Health, were obtained from the IEU Open-GWAS Project public database (25). Circulating metabolites were used as mediators to decompose the direct and indirect effects of hypothyroidism on OP through a two-step Mendelian randomization (TSMR) approach. The total effect of hypothyroidism on OP was denoted as TE, the effect of hypothyroidism on circulating metabolites as β1, and the effect of circulating metabolites on OP as β2. The indirect effect (IE), representing the causal pathway through which hypothyroidism influences the occurrence of OP via the mediator, was estimated using the product of coefficients method (β1 × β2). Accordingly, the proportion of the total effect mediated by the circulating metabolites was calculated as “indirect effect/total effect (IE/TE),” with the direct effect (DE) determined as TE - IE (26). Confidence intervals (CIs) for the mediated proportions were calculated using the delta method (27).

## 3 Results

### 3.1 Genetic variation selection

In this study, based on the selection criteria for instrumental variables, SNPs without a palindromic structure, no linkage disequilibrium, and an *F*-statistic >10 were selected. Specifically, 104, 11, 6, 60, and 161 SNPs were selected as valid instrumental variables (IVs) for hypothyroidism, hyperthyroidism, FT3, FT4, and TSH, respectively.

### 3.2 Causal effect of thyroid disease on OP

To investigate the effects of thyroid disease and thyroid function on OP, a TSMR analysis was conducted. Specifically, we examined the causal relationships between hypothyroidism, hyperthyroidism, FT3, FT4, TSH, and the risk of OP.

**Figure 2:**
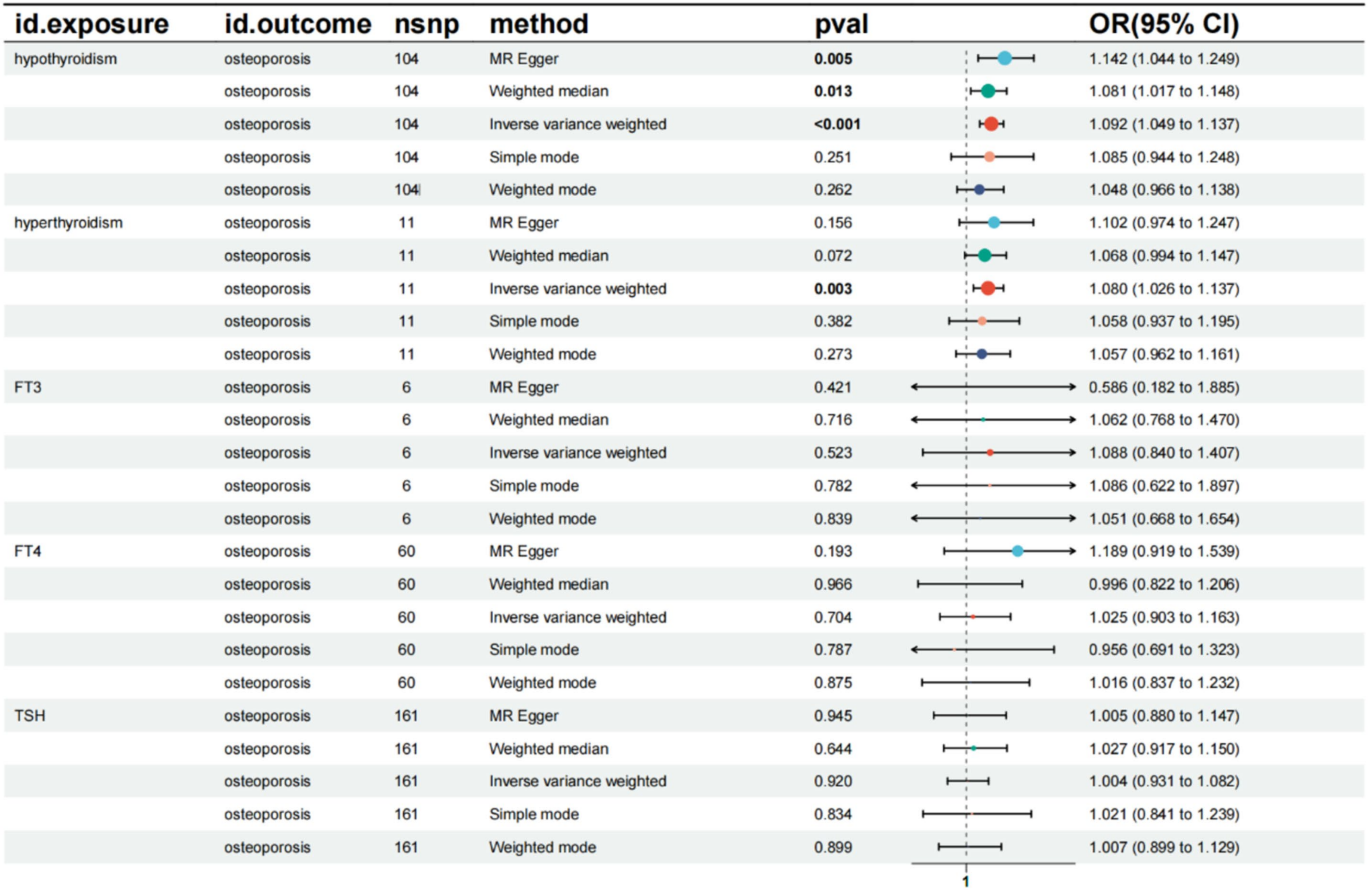
Summary of the five Mendelian randomization analysis methods. CI, confidence interval; OR, odds ratio; TSH, Thyroid stimulating hormone.

**Figure 3:**
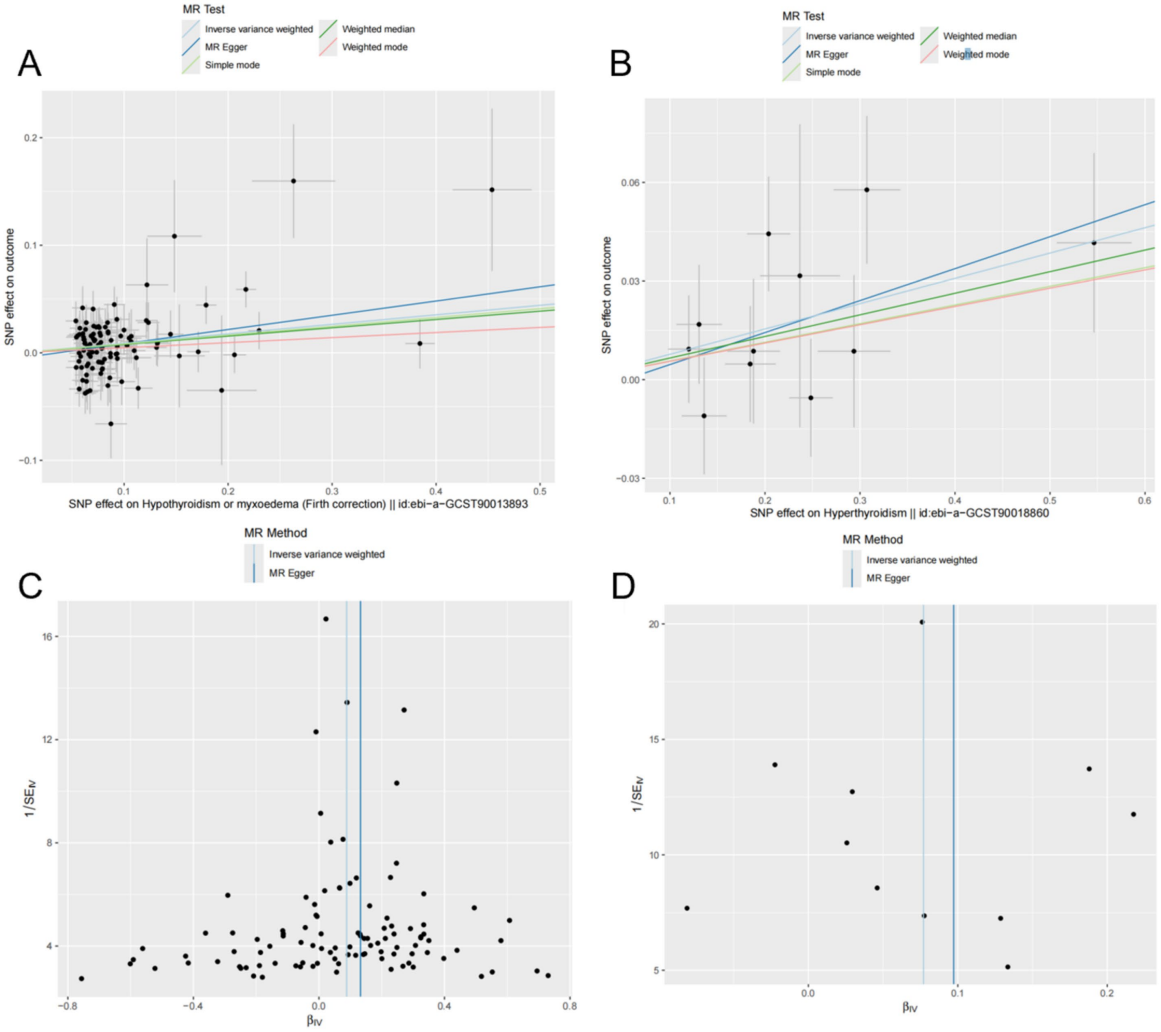
Mendelian randomization (MR) analyses of hyperthyroidism, hypothyroidism, and OP were conducted using multiple models. (A,B) Scatter plots depict the potential causal relationships between hypothyroidism (A), hyperthyroidism (B), and OP, where the slopes represent the estimated magnitude of causality. (C,D) Funnel plots present heterogeneity tests, highlighting causal effect estimates of hypothyroidism (C) and hyperthyroidism (D) on OP. The lines represent causal effect estimates derived from the inverse variance weighting and MR-Egger models.

**Figure 4:**
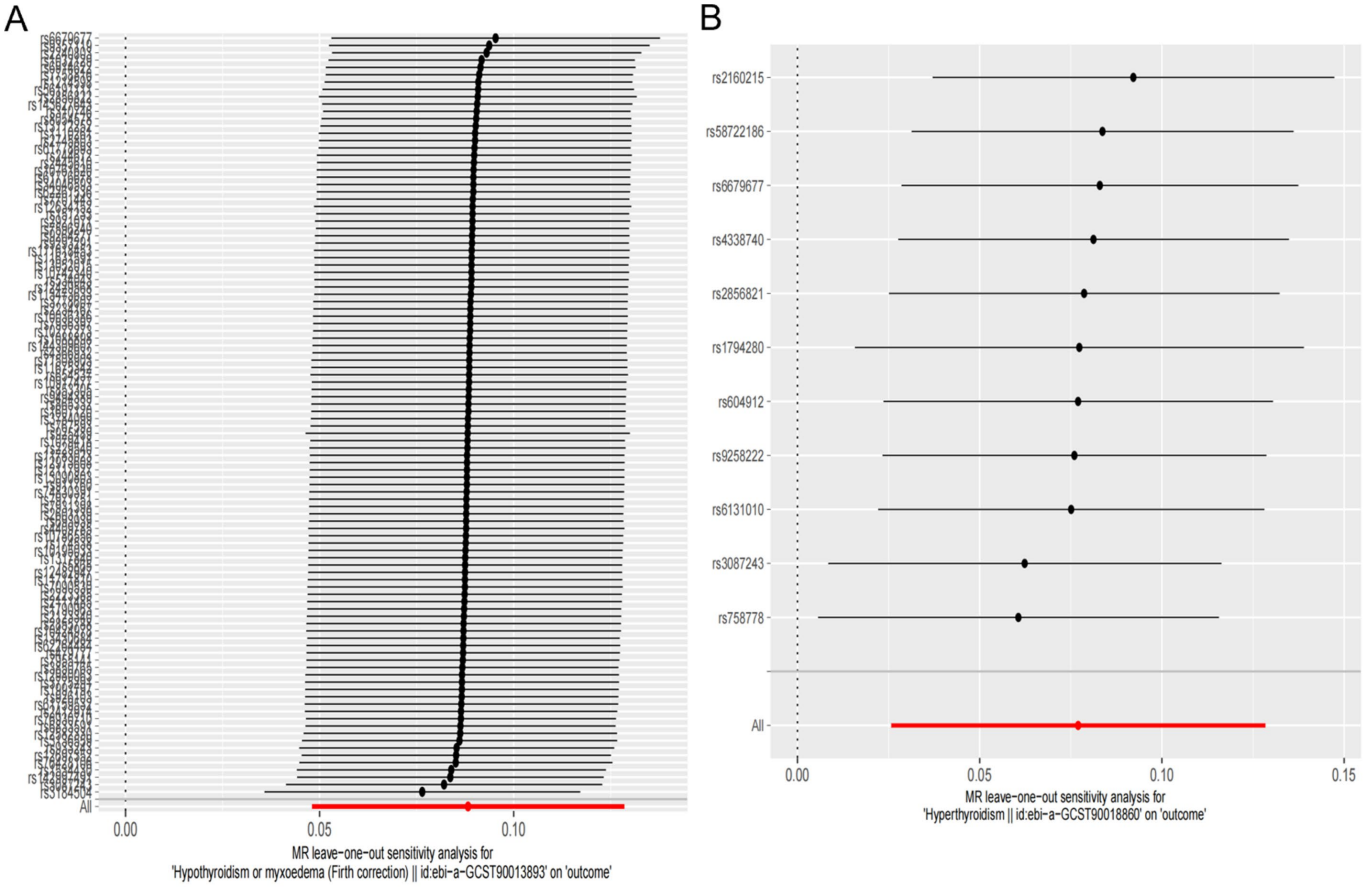
Plot of leave-one-out sensitivity analysis for hyperthyroidism and hypothyroidism.

The results indicated a causal relationship between hypothyroidism and an increased risk of OP (IVW: OR = 1.092, 95% CI: 1.049–1.137, *p* < 0.001). A causal relationship was also found between hyperthyroidism and OP risk (IVW: OR = 1.080, 95% CI: 1.026–1.137, *p* = 0.003). However, no causal relationships were observed between FT3, FT4, TSH, and OP risk (*p* > 0.05) (Figure 2). The intercept of the MR-Egger regression was not statistically significant, and the symmetry of the funnel plot around zero suggested that the selected instrumental variables were minimally, affected by horizontal pleiotropy (Figure 3). A multiplicity test conducted using R software showed that all *p*-values were greater than 0.05, indicating negligible or no multiplicity effects among the instrumental variables. Leave-one-out sensitivity analysis revealed that all SNPs in the hyperthyroidism and hypothyroidism groups were on one side of the distribution. The *p*-values from the heterogeneity test were greater than 0.05, indicating no biased SNPs (Figure 4).

### 3.3 Multivariate Mendelian analysis

**Figure 5:**
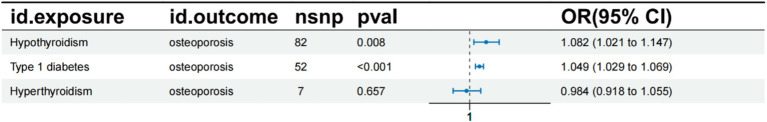
Multivariate Mendelian randomization analysis. CI, confidence interval; OR, odds ratio.

Diabetes mellitus is a known risk factor for OP and fractures, with patients with type 1 diabetes mellitus having an elevated risk of developing OP (28). Therefore, type 1 diabetes was included in this study for MVMR analysis. The results indicated that hyperthyroidism was no longer a risk factor for OP (OR = 0.984, 95% CI: 0.918–1.055, *p* = 0.657). However, hypothyroidism remained a risk factor for OP (OR = 1.082, 95% CI: 1.021–1.147, *p* = 0.008), and type 1 diabetes also emerged as a risk factor for OP (OR = 1.049, 95% CI: 1.029–1.069, *p* < 0.001) (Figure 5).

### 3.4 Mediated Mendelian randomization analysis

**Figure 6:**
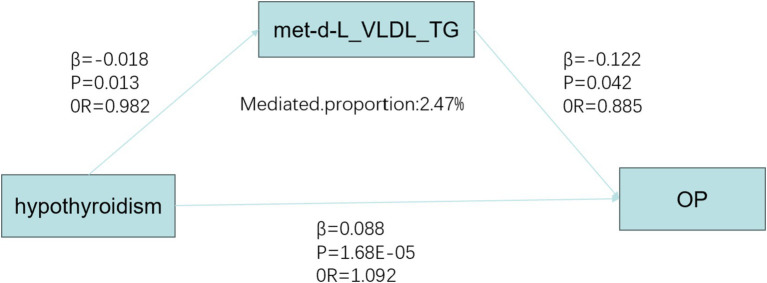
Mediated Mendelian analysis. OP, osteoporosis; OR, odds ratio; met-d-L_VLDL_TG, Triglycerides in large very low-density lipoprotein.

We investigated the effect of hypothyroidism on circulating metabolites in 249 individuals and identified a significant correlation between hypothyroidism and 59 circulating metabolites after applying FDR correction (*p* < 0.05) (29). Hypothyroidism was found to be a risk factor for citrate (OR = 1.021, 95% CI: 1.001–1.032, *p* = 0.005) and creatinine (OR = 1.018, 95% CI: 1.006–1.030, *p* = 0.017). It was also inversely associated with ApoA1 (OR = 0.980, 95% CI: 0.966–0.995, *p* = 0.038), cholines (OR = 0.976, 95% CI: 0.962–0.990, *p* = 0.011), the concentration of large high-density lipoprotein particles (HDL) (OR = 0.975, 95% CI: 0.960–0.990, *p* = 0.013), large cholesteryl esters in LDL (OR = 0.981, 95% CI: 0.965–0.996, *p* = 0.047), cholesteryl esters in large very-low-density lipoproteins (VLDL) (OR = 0.983, 95% CI: 0.970–0.997, p = 0.047), triglycerides in large VLDL (OR = 0.982, 95% CI: 0.969–0.996, *p* = 0.045), linoleic acid (LA) (OR = 0.970, 95% CI: 0.951–0.990, *p* = 0.018), low-density lipoprotein cholesterol (LDL) (OR = 0.981, 95% CI: 0.966–0.994, *p* = 0.032), free cholesterol in medium VLDL (OR = 0.981, 95% CI: 0.968–0.996, *p* = 0.039), concentration of medium VLDL particles (OR = 0.980, 95% CI: 0.968–0.993, *p* = 0.020), and phospholipids in small HDL (OR = 0.971, 95% CI: 0.960–0.985, *p* = 0.002), along with other protective factors. None of the selected SNPs exhibited significant horizontal pleiotropy (*p* > 0.05) or heterogeneity (*p* < 0.05). A Mendelian randomization analysis was then conducted with these 59 circulating metabolites as exposure variables and OP as the outcome variable. A causal effect was found only for triglycerides in large VLDL (OR = 0.885, 95% CI: 0.787–0.995, *p* = 0.042). Subsequently, a mediated Mendelian randomization analysis was performed, revealing that hypothyroidism exerted an indirect effect on OP through triglycerides in large VLDL, mediating 2.47% of the total effect (Figure 6).

## 4 Discussion

In this study, we employed univariate and MVMR analyses, along with mediation analyses, to evaluate the relationships between hypothyroidism, circulating metabolites, and OP. Our findings demonstrated that hypothyroidism and hyperthyroidism acted as risk factors for OP in two-sample MR analyses. However, after adjusting for confounders, including diabetes mellitus, in MVMR models, hyperthyroidism was no longer a significant risk factor. Furthermore, hypothyroidism may elevate the risk of OP by altering blood metabolite levels, with triglycerides in large VLDL potentially playing a mediating role, contributing to approximately 2.7% of the overall association between hypothyroidism and OP risk.

Thyroid hormone promotes the growth of long bones, stimulates the growth and differentiation of osteoblasts, and increases osteoclast activity (30). Yang et al. investigated the relationship between thyroid autoimmune diseases and OP within the normal range of TSH. Their results indicated that thyroid peroxidase antibodies (TPO) levels were significantly higher in the serum of patients with OP compared to those without the condition (*p* < 0.05) (31). TPO is a key indicator for diagnosing thyroid autoimmune disorders, which are most commonly associated with hyperthyroidism and hypothyroidism. These findings suggest a relationship between hyperthyroidism, hypothyroidism, and OP, which is consistent with the genetic conclusions drawn in this study. We propose that hypothyroidism is a risk factor for OP, a viewpoint shared by other scholars (32). Some studies have found that higher serum concentrations of TSH are associated with increased bone density and a lower likelihood of developing OP. The proposed mechanism is that TSH promotes osteoblast differentiation and proliferation. Additionally, a correlation has been identified between elevated FT3 and FT4 levels and an increased risk of fractures and reduced bone density (33). Studies have also shown that in older adults with normal TSH levels, a positive correlation between TSH concentration and OP still exists (34). In this study, from a genetic perspective, we did not find a definitive causal relationship between TSH and OP. This may be due to population selection bias in the data used, indicating the need for further research. Some scholars analyzed 3,338 men without thyroid or bone diseases and found no association between TSH, FT4, and either fracture or bone mineral density, which is consistent with our findings (35). In this study, we also included type 1 diabetes mellitus in the multivariate Mendelian analysis and found that both hypothyroidism and type 1 diabetes mellitus were risk factors for OP, though the underlying mechanism remains unclear. A possible explanation is a synergistic effect between hypothyroidism and type 1 diabetes.

Mediated MR approaches enable the investigation of potential causal pathways linking genetic risk factors to disease outcomes via specific biomarkers or metabolic pathways. In this study, we employed the genetic instrumental variable for hypothyroidism to evaluate its indirect influence on OP risk, mediated through circulating metabolites. We found that hypothyroidism may have an indirect effect on OP via triglycerides in large VLDL, though the underlying mechanism remains unknown. In a study involving patients in the early post-thyroidectomy phase, total cholesterol (TC), low-density lipoprotein cholesterol (LDL-C), triglycerides (TG), and the TC/HDL-C ratio were elevated in the thyroid hormone-naïve group compared to the control group (36). Cai et al. (37) analyzed 27 pregnant women with hypothyroidism and 28 healthy pregnant women, and found that plasma lipid levels in the hypothyroid group were significantly higher than in the healthy group. Zhang et al. (38) conducted a TSMR analysis and identified a causal relationship between dyslipidemia and OP, where VLDL was found to be a protective factor for OP (OR = 0.948), consistent with our findings. They further suggested that this result might be related to abnormal methylation gene modifications. In a study investigating the relationship between triglyceride-glucose (TyG) index and bone mass, findings indicated a significant association between elevated TyG and an increased risk of OP (39). Elevated triglycerides (TG) in VLDL may disrupt the balance between bone formation and resorption, but further research is required to elucidate the precise role of these metabolites in bone health.

Our study has several strengths, including multiple data sources, a large sample size, and the use of heterogeneity, multiplicity, sensitivity analyses, and FDR correction to ensure the robustness of the findings. Notably, this study is the first to use circulating metabolites as mediators to investigate the indirect effect of hypothyroidism on OP, the first to explore the causal effect of FT3 on OP, and the first to examine the impact of MVMR analyses on type 1 diabetes mellitus and thyroid disease in relation to the causal effect on OP.

Limitations of this study include: first, the study population was European, which may not be representative of the global population. Second, the only intervening variable analyzed was type 1 diabetes mellitus, while many other factors contribute to the development of OP, suggesting that future studies should incorporate additional variables for multivariate analysis. Third, due to the lack of distinction between OP subtypes in publicly available GWAS data on functional outcomes in hypothyroidism, it is currently not feasible to evaluate functional outcomes across specific OP subtypes. Fourthly, we only used summary data and did not assess and analyze other factors such as different age and gender. Moreover, full-scale clinical trials are necessary to validate these clinical conclusions. Therefore, a more comprehensive GWAS database and further trials are needed to clarify the mechanisms by which thyroid disease influences OP through lipid metabolism.

## 5 Conclusion

In this study, we evaluated the causal relationships between hypothyroidism, hyperthyroidism, FT3, FT4, TSH, and OP using TSMR analysis. Our findings suggest that hypothyroidism is a risk factor for OP, while a causal relationship between FT3, FT4, TSH, and OP has not yet been supported, and whether hyperthyroidism is a risk factor for OP requires further investigation. We also conducted a mediation analysis and found that hypothyroidism may have an indirect effect on OP through triglycerides in large VLDL, with a mediation proportion of 2.47%. This study highlights the importance of reminding patients with thyroid disease, especially those with concomitant dyslipidemia, and clinicians that lipid control should be emphasized in the treatment of thyroid disorders. Additionally, proactive measures should be taken to prevent the development and progression of osteoporosis.

## Data Availability

The datasets presented in this study can be found in online repositories. The names of the repository/repositories and accession number (s) can be found in the article/Supplementary material.
